# Does an Antibiotic Stewardship Applied in a Pig Farm Lead to Low ESBL Prevalence?

**DOI:** 10.3390/antibiotics10050574

**Published:** 2021-05-13

**Authors:** Claudine Fournier, Patrice Nordmann, Olivier Pittet, Laurent Poirel

**Affiliations:** 1 Medical and Molecular Microbiology Unit, Faculty of Science and Medicine, University of Fribourg, 1700 Fribourg, Switzerland; claudine.fournier@unifr.ch; 2 INSERM European Unit (IAME, France), University of Fribourg, 1700 Fribourg, Switzerland; patrice.nordmann@unifr.ch; 3 Swiss National Reference Center for Emerging Antibiotic Resistance (NARA), University of Fribourg, 1700 Fribourg, Switzerland; 4 Institute for Microbiology, University Hospital Centre, 1011 Lausanne, Switzerland; 5 Agricultural Institute of the Canton of Fribourg, Grangeneuve, 1725 Posieux, Switzerland; Olivier.Pittet@fr.ch

**Keywords:** ESBL, colistin, mobilized colistin resistance, pigs

## Abstract

*Background.* The aim of the present study was to prospectively evaluate the prevalence of intestinal carriage of colistin-resistant and extended-spectrum β-lactamase (ESBL)-producing *Enterobacterales* among pigs from a Swiss farm attending an animal health and antibiotic stewardship program and to determine the associated mechanisms of resistance. *Materials/Methods*. Eighty-one fecal samples were recovered and screened for either β-lactam-resistant, colistin-resistant, or aminoglycoside-resistant *Enterobacterales*, using respective screening media. All recovered isolates were tested for antimicrobial susceptibility and their clonal relationship (PFGE and MLST). Plasmid typing was performed by plasmid-based replicon typing (PBRT). Resistance genes were searched by PCR and sequencing. *Results.* A total of 38 ESBL-producing *Escherichia coli* and a single ESBL-producing *Enterobacter cloacae* were recovered from 81 pigs, corresponding to a prevalence of 50%, no other β-lactamase producer being identified. Among the 38 ESBL-producing *E. coli*, all belonged to sequence type (ST) ST10, except two ST34 and ST744 isolates. Among the ST10-*bla*_CTX-M-1_ isolates, three subclones (*n* = 22, *n* = 13, and *n* = 1, respectively) were identified according to the PFGE analysis. The most commonly identified IncI1 plasmid harboring the *bla*_CTX-M-1_ gene was 143 kb in size and coharbored other resistance genes. Only three colistin-resistant *Enterobacterales* isolates were recovered, namely two *Klebsiella pneumoniae* isolates and a single *E. cloacae* isolate. Screening for the plasmid-borne *mcr-1* to *mcr-9* genes in these three isolates gave negative results. The two *K. pneumoniae* isolates were clonally related, belonged to ST76, and harbored a truncated *mgrB* chromosomal gene being the source of colistin resistance. *Conclusion.* A high prevalence of fecal carriage of ESBL-producing *E. coli* was found, being mainly caused by the spread of a clonal lineage within the farm. By contrast, a low prevalence of colistin-resistant *Enterobacterales* was found.

## 1. Introduction

Antibiotic resistance is now considered a major public health concern, since it is nowadays on a rising trend, impacting the whole world and leading to difficult-to-treat infections. Moreover, recent perspectives indicate that the problem of antibiotic resistance shall be considered as a One Health issue, since the rising trend observed in human medicine is also observed in veterinary medicine and to a larger extent in the environment, especially among Gram-negatives [1]. Therefore, controlling the spread of multidrug resistance in one of these compartments might influence the others [2].

Food-producing animals, particularly swine, are known to be important reservoirs of antibiotic-resistant *Enterobacterales* and potential sources of transmission to humans [3,4]. In Switzerland, antibiotics, particularly β-lactams, may be prescribed to swine as therapeutics in case of infections. Hence, the microbiota of swine animals can be subjected to antibiotic selective pressure, and these animals may subsequently be colonized with multiresistant bacteria.

The aim of our study was to prospectively screen pigs from a Swiss farm for colonization by antibiotic-resistant enterobacterial isolates and to decipher the corresponding resistance mechanisms. A specific focus was made on β-lactams, particularly cephalosporins, since they are the most common antibiotics given for swine production. In addition, even if they are not part of the list of antibiotics given in that context, particular focus was placed on the occurrence of resistance to carbapenems (not given in animals), aminoglycosides, and polymyxins, as they represent crucial antibiotics in human medicine.

Rectal swabs were collected from healthy animals in a Swiss pig farm following the guidelines of SuisSano, a Swiss program for Animal Health from SuisAG since 2013 [5]. This program expects the farmer to record every antibiotic treatment of each pig, to use antimicrobial treatments as little as possible, and to strictly follow veterinary advice with respect to antibiotic usage.

## 2. Material and Methods

### 2.1. Swabs Recovery

The 81 fresh rectal swabs were recovered in M40 Amies media (Copan, Brescia, Italy) from healthy pigs on a farm in the Fribourg Canton (Switzerland). Swine were separated into ten stable boxes: three breeding sows with piglets in one box each, three boxes of weaning pigs (two with pigs from 18 to 20 kg and one from 9 to 10 kg), three boxes of fattening pigs, and a single reproduction box with 10 sows. Pigs had no contact with the outside environment and were all born on-site. All data concerning the type of livestock per box are provided in Table 1.

### 2.2. Selection of Resistant Isolates

Rectal swabs were cultured two hours after collection in Luria–Bertani broth (LBB, Roth, Karlsruhe, Germany) overnight. After LBB enrichment, a 10 µL loop of each broth was struck onto four different selective media, namely ChromID ESBL (bioMérieux, La Balme-La-Grotte, France) to select extended-spectrum β-lactamase (ESBL)-producing Gram-negatives, ChromID Carba Smart (bioMérieux) to select carbapenem-resistant Gram-negatives, home-made SuperAminoglycoside supplemented with amikacin 30 mg/L and gentamicin 30 mg/L to select pan-aminoglycoside-resistant Gram-negatives, and SuperPolymyxin supplemented with colistin 3.5 mg/L (EliTech Microbio, Signes, France) to select colistin-resistant Gram-negatives [6,7]. Grown colonies were tested by oxidase test (bioMérieux), and positive results were excluded as nonfermenting Gram-negative bacteria.

### 2.3. Identification and Phenotype of Resistance of Isolates

Oxidase negative grown colonies were first tested by rapid in-house tests, namely the Rapid ESBL NP, Rapid Carba NP, or Rapid Polymyxin NP tests to confirm the resistance phenotype of each isolate [8,9,10]. Isolates were then identified at the species level by using the API 20E system (bioMérieux). In case of difficult biochemical identification, 16S rRNA PCR amplification was performed using boiled crude DNA extracts of the tested isolate, and with primers 8F (5′-AGAGTTTGATCCTGGCTCAG-3′) and 1489R (5′-TACCTTGTTACGACTTCA-3′) using an annealing temperature of 55 °C. Sequencing and Blast analysis of the sequence were performed using the NCBI website to identify the species. Disk diffusion assays (Bio-Rad, Crissier, Switzerland) on Mueller–Hinton agar (Roth) square plates were performed to evaluate the phenotype of each isolate and to determine the susceptibility to antibiotics, including amoxicillin, ticarcillin, piperacillin, temocillin, amoxicillin + clavulanic acid, ticarcillin + clavulanic acid, piperacillin + tazobactam, cephalothin, cefotaxime, ceftazidime, cefoxitin, cefepime, aztreonam, ertapenem, meropenem, imipenem, nalidixic acid, ciprofloxacin, tetracycline, tigecycline, chloramphenicol, fosfomycin, sulfonamide, sulfamethoxazole-trimethoprim, gentamicin, amikacin, tobramycin, and kanamycin, while susceptibility to colistin was tested by broth microdilution, according to EUCAST recommendations [11].

### 2.4. Identification of β-Lactamase Genes

Production of an ESBL was firstly assessed by using the Rapid ESBL, and then PCR amplification was performed on ESBL-positive isolates to identify the corresponding β-lactamase genes as previously described [12]. Sequencing of the obtained amplicons and in silico Blast analyses were performed to identify the exact nature of the β-lactamase gene (Microsynth, Balgach, Switzerland) [13].

### 2.5. Genetic Subtyping

Clonality of each isolate was evaluated by comparison of the DNA fingerprints obtained by pulsed-field gel electrophoresis (PFGE) according to CDC. Multilocus sequence typing (MLST) was performed on a representative isolate of each PFGE pattern. This method consists of the amplification of seven housekeeping genes (*adk*, *fumC*, *gyrB*, *icd*, *mdh*, *purA*, and *recA*) and sequencing. Identification of the allele of each gene is done by query of each sequence in PubMLST database (www.pubmlst.org) and MLST allelic profiles are determined according to Achtmann MLST scheme [14,15].

### 2.6. Plasmid Characterization

Plasmids carrying the *bla*_CTX-M-1_ were first extracted by using the Kieser method [16]. Purified plasmids from each isolate were then transformed in the recipient *E. coli* strain TOP10 by electroporation using a MicroPulser electroporator (Bio-Rad). Transformants were selected on a Luria–Bertani agar plate supplemented with 1 µg/mL of cefotaxime. PCR amplification of the *bla*_CTX-M-1_ gene was performed on the transformants in order to confirm the success of the transformation [12]. In order to identify the plasmid bearing the *bla*_CTX-M-1_ gene, PCR-based replicon typing (PBRT) using IncI1, IncF, IncHI1, and IncN primers were performed [17]. PCR was completed using DNA extracts from the *E. coli* transformants and in-house sequenced positive controls to determine the incompatibility group of the plasmids [18].

Plasmid extracts from *E. coli* transformants harboring representative plasmids bearing the gene *bla*_CTX-M-1_ were sequenced by Illumina for short reads and Nanopore for long reads. DNA library for Nanopore and Illumina sequencing was prepared using the NEBNext End Repair/dA-tailing module (Ipswich, MA, USA) and the ligation sequencing kit Nanopore SQK-LSK109 and Nextera XL library kit, respectively. The obtained plasmid data sequences in fastQ were assembled and polished by CLC Main Genomic Workbench version 20.04 from Qiagen (Hilden, Germany). Annotation of the plasmid sequences was performed by CARD and analysis by PlasmidFinder [19,20].

## 3. Results

### 3.1. Prevalence of β-Lactamase-Producing and Colistin-Resistant Enterobacterales

A total of 39 ESBL-producing *Enterobacterales* was recovered from the 81 rectal swabs. Among them, 38 *E. coli* and a single *E. cloacae* were identified, all of them being positive for the *bla*_CTX-M-1_ gene. These ESBL producers were recovered from all the ten stable boxes from which samples had been recovered (Table 1 and Table 2).

Three colistin-resistant *Enterobacterales* were recovered onto the SuperPolymyxin medium, two being *K. pneumoniae* and one being *E. cloacae*. One *K. pneumoniae* was found in a weaning pig of ca. 20 kg, whereas the single *E. cloacae* and the other colistin-resistant *K. pneumoniae* were recovered from pigs living in the same stable (Table 2).

### 3.2. Antimicrobial Resistance Features of Colistin-Resistant Isolates

Colistin susceptibility testing showed that the isolates were resistant to colistin with MICs of 64, 32, and 128 mg/L, respectively, but remained susceptible to β-lactams. PCR screening of *mcr-1* to *mcr-9* gave negative results for the three strains. Two *K. pneumoniae* isolates harbored a chromosomal mutation leading to a premature stop codon (C28*) in the *mgrB* gene sequence, which was, therefore, the source of the acquired resistance as previously shown [6].

### 3.3. Clonal Relationship

**Figure 1:**
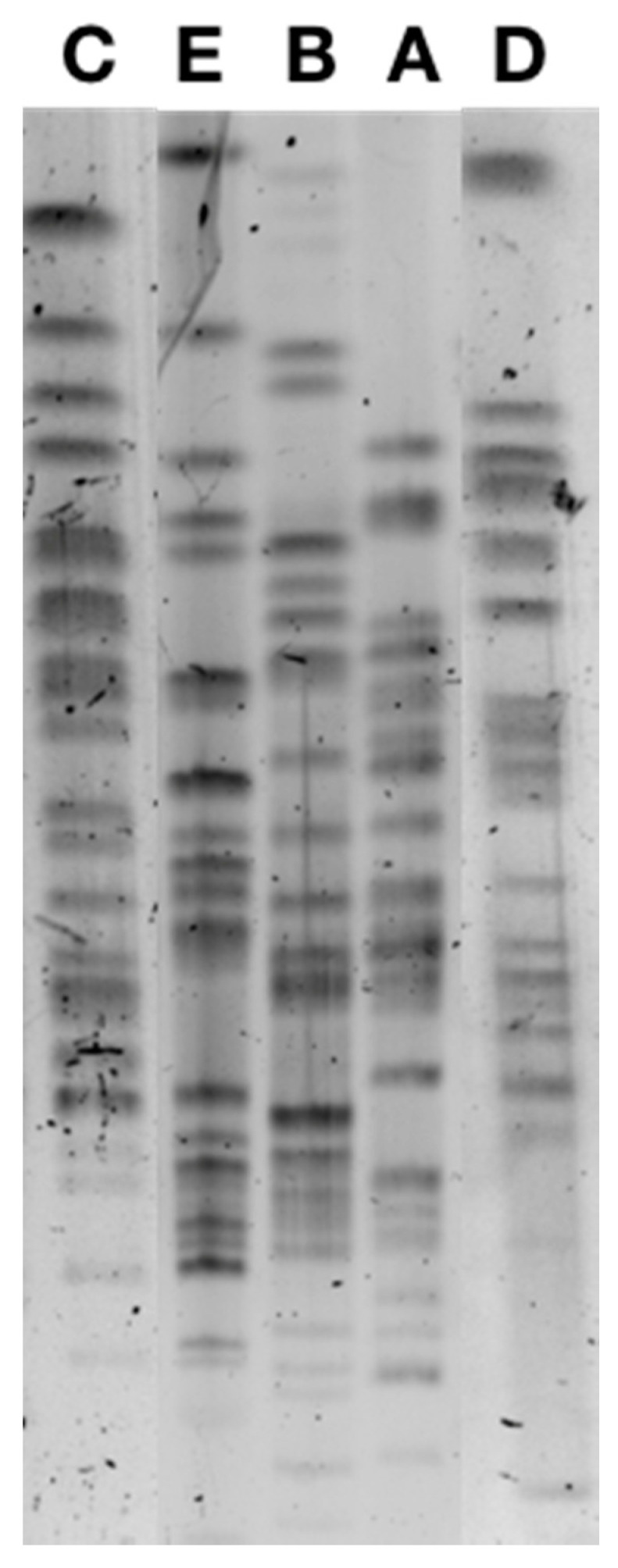
Pulsed-field gel electrophoresis of CTX-M-1-positive *E. coli* isolates corresponding to pulsotypes A, B, C, D, and E.

PFGE analysis identified five different pulsotypes among the 38 ESBL-producing *E. coli* (Figure 1), the majority being from the sequence type (ST) ST10 and two isolates being ST34 and ST744, respectively, and also belonging to clonal complex CC10. Pulsotype A included 22 isolates, pulsotype B included 13 isolates, and pulsotype C included 1 isolate, all belonging to ST10, while the 2 ST34 and ST744 isolates corresponded to pulsotypes C and D (Table 2). Pulsotype B was divided in sub-pulsotypes B1 (12 isolates) and B2 (1 isolate) according to the antibiotic co-resistances observed.

The two colistin-resistant *K. pneumoniae* showed a different pulsotype although both belonged to the same sequence type (ST76).

### 3.4. Plasmid Analysis

Transformants showing an ESBL phenotype were obtained from each pulsotype. PBRT analysis on the transformants showed that the *bla*_CTX-M-1_ was always located on an IncI1-type plasmid. Whole-plasmid sequencing was performed for those plasmids being the most prevalent, namely the two recovered from isolates belonging to pulsotypes A and B, respectively. Plasmid sequences of plasmids pCTX-M-1.A and pCTX-M-1.B1 have been deposited in GenBank under accession numbers MW978788 and MW978789, respectively. Sequence analysis revealed that both plasmids belong to the incompatibility group IncI1-I***γ*** and belonged to pMLST ST3 type. Those two plasmids (namely pCTX-M-1.A and pCTX-M-1.B1) were 145,152 bp and 111,787 bp in size, respectively. Both plasmids revealed a similar backbone structure also found on the IncI1 reference plasmid R64 (GenBank accession number AP005147.1). Interestingly, these plasmids harbored the *doc* and *phd* toxin–antitoxin system genes, involved in plasmid maintenance, and the *psiA* and *psiB* SOS inhibition genes. Those plasmids bearing the *bla*_CTX-M-1_ gene coharbored the *sul2* and *tetA* genes, respectively encoding resistance to sulfonamides and tetracycline.

## 4. Discussion

In this study, a high prevalence of ESBL-producing *Enterobacterales* was found among pigs from a livestock farm in Switzerland, with 50% of the animals being colonized by ESBL-producing isolates, although only 4% were colonized with colistin-resistant *E. coli* or *K. pneumoniae* isolates. Neither carbapenem-resistant nor pan-aminoglycoside-resistant isolates could be recovered upon selection with respective selective media.

The rate of ESBL colonization was heterogeneous among the different stables (see Table 1).

This high rate of ESBL producers corresponded to an unexpected result. Indeed, such prevalence of ESBL-producing *Enterobacterales* was not expected in a farm with low antibiotic usage. To our knowledge, this is the first epidemiological report of acquired broad-spectrum cephalosporin resistance in a pig farm following such a struct antibiotic stewardship.

By contrast, the low rate of colistin-resistant isolates correlated with the lack of colistin usage for any purposes, including any food supplementation, and was in accordance with previous studies [12,21]. The three colistin-resistant isolates were negative for *mcr* genes, exhibiting nontransferable chromosomally encoded resistance mechanisms. It is worth noting that these three colistin-resistant *K. pneumoniae* were of ST76, an ST previously identified among NDM- or KPC-type carbapenemase producers recovered from humans in China [22,23,24]. This observation raises some concern about the epidemiology of such multidrug-resistant ST76 *K. pneumoniae*, strongly supporting the strict monitoring and prevention of its spread. In addition to a recent report, the low-level colistin resistance rate (3.7%) evidenced in our study further highlights the benefit of banning colistin use in livestock [12].

Although evaluation of the clonality of the ESBL-producing *E. coli* revealed two major clones, they actually corresponded to a single sequence type, namely ST10, harboring a similar plasmid IncI1-I***γ*** harboring *tetA* and *sul2* antibiotic resistance genes in both cases (Table 2). The high cephalosporin resistance rate observed here was therefore mainly due to the spread of clonal strains rather than the dissemination of epidemic plasmids. It is noteworthy that very low rates of coresistance to fluoroquinolones were observed among those ESBL producers, in contrast to what is being observed among human isolates.

*E. coli* ST10 has been previously reported in animals and in humans [25]. IncI1-I***γ***-type plasmids harboring *bla*_CTX-M-1_ have already been identified not only in different food-producing animals including broilers, poultry, and cattle, but also in the environment (rivers) and in both community patients and healthy people in Switzerland and around the world, being, therefore, a common *E. coli* background whose reservoir is hypothesized to be mainly animal [17,26,27,28].

The high rate of CTX-M-1 producers (actually being commonly identified in animals) among fattening pigs and sows would suggest the presence of selective pressure in the farm. However, the absence of antibiotic usage in these fattening pigs did not support an antibiotic-related one. The maintenance of the CTX-M-1-encoding plasmid might, however, be explained by the presence of a toxin–antitoxin system [29,30,31,32]. The presence of toxin–antitoxin systems on plasmids is known to be responsible for the postsegregational killing of daughter cells that do not contain the plasmid after the cell division completion [33]. On the other hand, the PsiAB proteins are known to allow a successful transfer of plasmid genetic information during conjugation by inhibiting the SOS system of the receptor bacteria, hence favoring their dissemination. Both systems indicate that the plasmid IncI1 pMLST3 identified here may be considered as a successful plasmid in terms of maintenance within a population and of dissemination. Nevertheless, our observations require further investigations to provide possible explanations about such an occurrence.

## Figures and Tables

**Table 1:** Rate and features of resistance isolates distribution in pig stables.

Stable Number	Number of Pigs	Number of Resistant Isolates	Type of Livestock	Weight of Piglets	Clones Present	Rate of Pigs Carrying Resistant Strains	Resistant Determinant	Co-Resistance Phenotype
1	8	7	Fattening	40 to 42 kg	A (*n* = 6)	87.50%	*bla*_CTX-M-1_	SUL, TET ^a^ (*n* = 1); SUL (*n* = 4);
								None (*n* = 1)
					C (*n* = 1)		*bla*_CTX-M-1_	NAL, CIP, TET, SUL, CHL, FLO (*n* = 1)
2	8	5	Fattening	40 to 42 kg	A (*n* = 5)	62.50%	*bla*_CTX-M-1_	SUL (*n* = 4); NAL, CIP, TET, SUL, CHL, FLO (*n* = 1)
3	6	2	Fattening	102 kg	A (*n* = 1)	33.30%	*bla*_CTX-M-1_	SUL (*n* = 1)
					B1 (*n* = 1)		*bla*_CTX-M-1_	SUL (*n* = 1)
4	10	4	Weaning	18 to 20 kg	A (*n* = 4)	40%	*bla*_CTX-M-1_	SUL (*n* = 4)
5	10	8	Weaning	18 to 20 kg	A (*n* = 3)	60%	*bla*_CTX-M-1_	SUL (*n* = 3)
					B1 (*n* = 2)		*bla*_CTX-M-1_	SUL, TET (*n* = 2)
					E (*n* = 1)		*bla*_CTX-M-1_	TET (*n* = 1)
					*E. cloacae* (*n* = 1)		*bla*_CTX-M-1_	SUL, TET (*n* = 1)
					*K. pneumoniae* (*n* = 1)		*mgrB* truncation	SUL (*n* = 1)
6	20	4	Weaning	9 to 10 kg	A (*n* = 2)	20%	*bla*_CTX-M-1_	SUL (*n* = 1); NAL, CIP, SUL, TET, CHL, FLO (*n* = 1);
					*E. cloacae* (*n* = 1)		ND ^d^	None (*n* = 1)
					*K. pneumoniae* (*n* = 1)		*mgrB* truncation	None (*n* = 1)
7	10	8	Reproduction	-	B1 (*n* = 7)		*bla*_CTX-M-1_	SUL, TET (*n* = 7)
					D (*n* = 1)		*bla*_CTX-M-1_	GMI, KMN, TMN, SUL, TET (*n* = 1)
8	3	2	Sow ^b^ with 3 piglets		B1 (*n* = 2)	66%	*bla*_CTX-M-1_	SUL, TET (*n* = 2)
9	3	2	Sow ^c^ with 3 piglets		B2 (*n* = 1)	33%	*bla*_TEM-1_	KMN, SUL, SXT, TET (*n* = 1)
					B1 (*n* = 1)	33%	*bla*_CTX-M-1_	SUL, TET (*n* = 1)
10	3	1	Sow ^d^ with 3 piglets		A (*n* = 1)	33%	*bla*_CTX-M-1_	SUL (*n* = 1)

^a^ Antibiotic abbreviations: CHL—chloramphenicol; CIP—ciprofloxacin; FLO—florfenicol, SUL—sulfonamide; SXT—trimethoprime-sulfamethoxazol; GMI—gentamicin; KMN—kanamycin; NAL—nalidixic acid; TMN—tobramycin; TET—tetracycline. ^b^ Sow treated with 3 doses of Betamox (amoxicillin, 15 mg/kg, iv). ^c^ Sow treated with 2 doses of Borgal (sulfadoxin-trimethoprim 24%, 8–12 mL, iv) and Rifen (cetoprofen 10%, 3 mL/100 kg, iv). ^d^ Resistance to colistin, mechanism not determined. ND: not determined.

**Table 2:** Phenotype and genetic features associated with the ESBL-positive isolates.

Number of Isolates	Species	Phylogenic Group	ST	Pulsotype	Resistance Determinants	Incompatibility Group of the Plasmid Carrying *bla*_CTX-M-1_	Coresistance on the Plasmid Carrying *bla*_CTX-M-1_
22	*E. coli*	A	ST10	A	*bla*_CTX-M-1_	Inc I1	TET, SUL ^a^
12	*E. coli*	A	ST10	B1 ^b^	*bla*_CTX-M-1_	Inc I1	TET, SUL
1	*E. coli*	A	ST10	B2 ^b^	*bla*_CTX-M-1_	IncI1	GMI, KMN, TMN, SUL, TET
1	*E. coli*	A	ST744	C	*bla*_CTX-M-1_	Inc I1	TET, SUL
1	*E. coli*	A	ST34	D	*bla*_CTX-M-1_	Inc I1	TET, SUL
1	*E. coli*	A	ST10	E	*bla*_CTX-M-1_	Inc I1	TET, SUL
1	*E. cloacae*		ND		*bla*_CTX-M-1_	Inc I1	TET, SUL

^a^ Antibiotic abbreviations: SUL—sulfonamides; TET—tetracycline; GMI—gentamicin; KMN—kanamycin; TMN—tobramycin; ND: not determined. ^b^ The pulsotype B was divided in B1 and B2 sub-pulsotypes according to co-resistance.

## Data Availability

The datasets used and/or analysed during the current study are available from the corresponding author on request.
